# Association of metabolically unhealthy non-obese and metabolically healthy obese individuals with arterial stiffness and 10-year cardiovascular disease risk: a cross-sectional study in Chinese adults

**DOI:** 10.1186/s12937-023-00870-9

**Published:** 2023-09-20

**Authors:** Wen Guo, Jue Jia, Mengyao Zhan, Xiaona Li, Wenfang Zhu, Jing Lu, Xin Zhao, Nainzhen Xu, Qun Zhang

**Affiliations:** 1 Department of Health Promotion Center, the First Affiliated Hospital with Nanjing Medical University, 300 Guangzhou Road, Nanjing, 210029 China; 2 Department of Endocrinology and Metabolism, the Affiliated Hospital of Jiangsu University, Zhenjiang, 212000 China; 3 Department of Epidemiology, Center for Global Health, School of Public Health, Nanjing Medical University, Nanjing, 211166 China

**Keywords:** Arterial stiffness, 10-year CVD risk, Metabolic syndrome, Obesity

## Abstract

**Background:**

The relationship between metabolically healthy obese individuals (MHO) and cardiovascular disease (CVD) risk is disputed. This study investigated the association of metabolically unhealthy non-obese(MUNO) individuals and MHO with arterial stiffness and 10-year CVD risk.

**Methods:**

A total of 13,435 participants were enrolled and further divided into the metabolically healthy non-obese (MHNO) phenotype (n = 4927), MUNO phenotype (n = 1971), MHO phenotype (n = 2537) and metabolically unhealthy obese (MUO) phenotype (n = 4000) according to body mass index (BMI) and metabolic status. We used brachial ankle pulse wave velocity (baPWV) to measure arterial stiffness and the Framingham risk score (FRS) to evaluate the 10-year CVD risk.

**Results:**

The MUO and MUNO phenotypes had higher mean baPWV values than the MHO and MHNO phenotypes, regardless of age (1446.19 ± 233.65 vs. 1423.29 ± 240.72 vs. 1283.57 ± 213.77 vs. 1234.08 ± 215.99 cm/s, *P* < 0.001). Logistic regression analysis indicated that the MUNO and MUO phenotypes were independently correlated with elevated baPWV and 10-year CVD risk, while the MHO phenotype was independently associated with only the 10-year CVD risk. In metabolically healthy subjects, BMI showed a dose-dependent increase in the risk of elevated baPWV, with an adjusted OR of 1.007 (*95% CI* 1.004–1.010, *P* < 0.001). However, in metabolically unhealthy participants, the estimate for the relationship between elevated baPWV and BMI was nonsignificant.

**Conclusions:**

The MUNO phenotype exhibits increased arterial stiffness and 10-year CVD risk. However, BMI is positively and dose-dependently correlated with arterial stiffness only in metabolically healthy subjects. We speculate that metabolic status may be a strong confounder in the obesity–elevated baPWV association.

## Background

The prevalence of metabolic syndrome (MetS) has rapidly and substantially increased in recent decades, including in developing and developed countries, representing a considerable clinical burden and a public health concern. Its components, such as obesity, hyperglycemia, hypertension and hyperlipidemia, are all significantly correlated with cardiovascular disease (CVD) [1, 2]. A better understanding of the complex pathophysiological mechanisms of CVD caused by MetS would greatly benefit early intervention and treatment efforts.

An increasing number of studies have shown that being overweight and obesity are the strongest risk factors for MetS [3]. It is of great interest to find a specific subset of metabolic abnormalities presenting without obesity, referred to as metabolically unhealthy non-obesity (MUNO). Large-scale epidemiological studies have demonstrated that the MUNO phenotype is associated with an increased risk for cardiovascular events compared with non-obese subjects without metabolic syndrome (MHNO) [4, 5]. Moreover, previous studies showed that metabolically abnormal but normal weight individuals even had higher maximal carotid intima media thickness values than metabolically abnormal obese participants. Furthermore, normal weight with MetS participants had the highest risk of death from all causes and CVD during 10 years of follow-up [6, 7]. On the other hand, it is worth mentioning another specific subset of obesity with a relatively favorable metabolic profile, referred to as metabolically healthy obesity (MHO). Recently, MHO has evolved into a contentious subject of debate due to controversial results regarding whether it increases the risk of CVD. Some studies have demonstrated that MHO is not a stable or reliable indicator of future risk for CVD [8], and MHO subjects do not have higher all-cause and CVD mortality risk than MHNO subjects [9]. Other studies have concluded that MHO is a transient condition and is independently associated with elevated CVD risk [10, 11]. Considering the abovementioned research, the association of MHNO and MHO with brachial-ankle pulse wave velocity (baPWV), which has an independent prognostic value for CVD risk [12], is worthy of further exploration. Therefore, we investigated the association of MHNO and MHO with arterial stiffness using baPWV and the 10-year CVD risk evaluated by the Framingham risk score.

## Materials and methods

### Study population

The sample comprised 13,435 adult individuals who underwent baPWV measurements at the Health Promotion Center of the First Affiliated Hospital of Nanjing Medical University from September 2017 to January 2020. Each participant underwent a face-to-face interview by physicians. We excluded participants with histories of cardiovascular and cerebrovascular events, taking medications that affect lipid levels. Some conditions such as inflammatory conditions, malignancy, pregnancy, renal or hepatic dysfunction that affect the study results were also excluded. All eligible participants agreed to participate in this study and provided written informed consent. The Ethics Committee of the First Affiliated Hospital of Nanjing Medical University approved the study protocol used in this work.

### Physical examination and biochemical tests

Anthropometric measurements, including height, weight, and blood pressure, were taken by well-trained nurses according to standard protocols. Body mass index (BMI) was then calculated by dividing body weight (in kilograms) by the square of the body height (in meters). Blood samples were collected after an overnight fast of 12 h. All eligible participants received blood tests (including white blood cell count (WBCC), neutrophil count, lipid profile, fasting plasma glucose (FPG), glycated hemoglobin A1c (HbA1c), and uric acid levels). An automated blood cell counter (Beckman Coulter Ireland Inc. Mervue, Galway, Ireland) and a biochemical autoanalyzer (Chemistry Analyzer Au5800, Olympus Corporation, Tokyo, Japan) were used in this study.

### Measurement of baPWV

Before examination, participants had a minimum resting time of 5–10 min in a supine position. We used a VP-1000 automated PWV/ABI analyzer (Omron Colin BP-203RPE III, Omron Health Care, Kyoto, Japan) to measure bilateral baPWV. Briefly, baPWV was measured from the ascending point of the right brachial pulse volume recorder to the ascending point of each ankle pulse volume recorder. We adopted the mean value of the right and left baPWV for analysis. Due to the lack of a definite cutoff value of baPWV for arterial stiffness, we considered values higher than the cutoff level between the third and fourth quartiles (> 75th percentile) of baPWV of this study population as arterial stiffness, according to a previous study [13].

### Framingham 10-year risk estimation

The Framingham risk score (FRS) is the best known and most common tool to estimate CVD risk. It is calculated based on traditional risk factors, including sex, age, tobacco smoking, systolic blood pressure (SBP) (treated or untreated), total cholesterol (TC) and high-density lipoprotein cholesterol (HDL-C) [14]. The estimated risk for 10-year CVD was classified as low risk (< 10%), intermediate risk (10–20%), and high risk (> 20%).

### Definitions of weight and metabolic status

According to Asia-specific BMI criteria, obesity was defined as a BMI ≥ 25 kg/m^2^. A metabolically unhealthy individual was defined as an individual having two or more of the following components according to the National Cholesterol Education Program Adult Treatment Panel III: (1) elevated triglyceride (TG): serum TG level ≥ 150 mg/dL (1.70 mmol/L); (2) reduced high density lipoprotein-cholesterol (HDL-C): HDL-C level < 40 mg/dL (1.04 mmol/L) in men or 50 mg/dl (1.29 mmol/L) in women; (3) elevated blood pressure: systolic blood pressure (SBP) ≥ 130 mmHg and/or diastolic blood pressure (DBP) ≥ 85 mmHg or antihypertensive drug treatment; and (4) increased fasting blood glucose (FBG): serum glucose level ≥ 100 mg/dl (5.6 mmol/L) and/or the use of drug treatment for increased glucose. The waist circumference criterion was excluded due to its collinearity with BMI. By combining BMI and metabolic components, all participants were further divided into four phenotypes: [1] BMI < 25 kg/m2 and metabolically healthy status (MHNO); [2] BMI < 25 kg/m2 and metabolically unhealthy status (MUNO); [3] BMI ≥ 25 kg/m2 and metabolically healthy status (MHO); and [4] BMI ≥ 25 kg/m2 and metabolically unhealthy status (MUO) [15].

### Statistical analysis

Data were analyzed using SPSS 18.0 statistical software. Each variable was assessed for a normal distribution. Continuous variables were expressed as the mean ± standard deviation (SD) or median (inter-quartile range[25-75%]) and were compared using the one-way ANOVA or KruskaleWallis H test. Categorical variables were presented as numbers and proportions and were compared using the chi-square test. Multivariate logistic regression analyses were used to analyze the significance of variables in arterial stiffness and the 10-year CVD risk after adjusting for other confounding risk factors. The metabolic status interaction between BMI and baPWV was assessed using generalized linear models (GLMs). Significance tests were two-tailed, and a *p* value < 0.05 was considered significant.

## Results

### Baseline characteristics of the study population according to different phenotypes

Overall, 44.44% (n = 5971) of the participants were metabolically unhealthy, and 48.66% (n = 6537) were obese. The MUNO phenotype (n = 1971) and MHO phenotype (n = 2537) accounted for 14.67% and 18.88% of the total population, respectively. Participants with the MUO and MUNO phenotypes were older and had higher SBP, diastolic blood pressure (DBP), WBCC, neutrophil count, FPG, HbA1c, TC, triglyceride (TG), and low-density lipoprotein cholesterol (LDL-C) and lower HDL-C than those with the MHNO and MHO phenotypes. The percentage of males, smoking rates, and serum uric acid levels were higher in participants with the MHO phenotype than in those with the MHNO phenotype. Detailed results are listed in Table 1.

**Table 1:** Baseline characteristics of participants according to metabolic health and obesity

	n	Age (years)	Male (n, %)	Smoking (n, %)	BMI (kg/m2)	
MHNO	4927	47(41, 54)	1836(37.26)	594(12.05)	21.85 ± 1.75	
MHO	2537	48(42, 55)	1667(65.71)^a^	512(20.18) ^a^	26.62 ± 1.82 ^a^	
MUNO	1917	52(46, 58)^a,b^	980(49.72)^a,b^	344(17.45) ^a,b^	22.76 ± 1.40 ^a,b^	
MUO	4000	51(45, 57) ^a,b^	2969(74.22)^a,bc^	1071(26.77) ^a,bc^	27.46 ± 2.35 ^a,bc^	
	**SBP (mmHg)**	**DBP (mmHg)**	**WBCC (109/L)**	**Neutrophil count(109/L)**	**FPG (mmol/L)**	**HbA1c (%)**
MHNO	117(108,126)	71(65,78)	5.41 ± 1.37	3.15 ± 1.08	5.08 ± 0.56	5.41 ± 0.41
MHO	122(114, 132) ^a^	76(70, 82) ^a^	5.78 ± 1.48 ^a^	3.33 ± 1.16 ^a^	5.16 ± 0.58 ^a^	5.48 ± 0.44 ^a^
MUNO	132(121,141) ^a,b^	80(72,87) ^a,b^	5.87 ± 1.49 ^a^	3.45 ± 1.15 ^a,b^	5.94 ± 1.71 ^a,b^	5.81 ± 0.96 ^a,b^
MUO	135(126, 146) ^a,bc^	85(77, 91) ^a,bc^	6.19 ± 1.52 ^a,bc^	3.60 ± 1.17 ^a,bc^	6.08 ± 1.57 ^a,bc^	5.91 ± 0.94 ^a,bc^
	**TC (mmol/L)**	**TG (mmol/L)**	**LDL-C (mmol/L)**	**HDL-C (mmol/L)**	**Uric acid (umol/l)**	
MHNO	5.28 ± 1.00	1.04 (0.81, 1.34)	3.20 ± 0.74	1.51 ± 0.30	285(242,342)	
MHO	5.32 ± 0.99	1.26(1.00, 1.54) ^a^	3.34 ± 0.75 ^a^	1.34 ± 0.24 ^a^	344(287, 403) ^a^	
MUNO	5.46 ± 1.11 ^a,b^	1.85(1.35, 2.48) ^a,b^	3.42 ± 0.79 ^a,b^	1.25 ± 0.28 ^a,b^	319(271,378) ^a,b^	
MUO	5.48 ± 1.14 ^a,b^	2.13(1.62, 2.96) ^a,bc^	3.46 ± 0.79 ^a,b^	1.16 ± 0.24 ^a,bc^	371(314, 429) ^a,bc^	

MHNO, metabolically healthy non-obese; MHO, metabolically healthy obese; MUNO, metabolically unhealthy non-obese; MUO: metabolically unhealthy obese; BMI, body mass index; SBP, systolic blood pressure; DBP, diastolic blood pressure;

WBCC, white blood cell count; FPG, fasting plasma glucose; HbA1c, glycated hemoglobin A1c; TC, total cholesterol; TG, triacylglyceride; LDL-C, low-density lipoprotein cholesterol; HDL-C, high density lipoprotein cholesterol. Compared with MHNO, ^a^*P*<0.05; Compared with MHO, ^b^*P*<0.05; Compared with MUNO, ^c^*P*<0.05

### Comparison of baPWV and 10-year CVD risk among different phenotypes

**Fig. 1:**
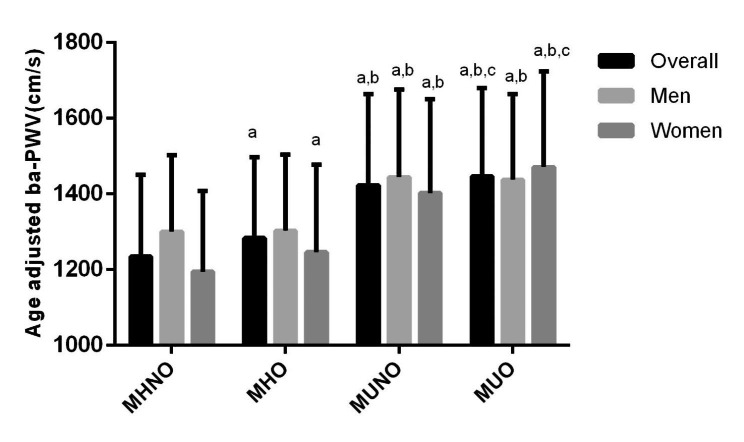
Age adjusted baPWV compared among different phenotypes in all participants, men, and women. Compared with MHNO, ^a^*P*<0.05; Compared with MHO, ^b^*P*<0.05; Compared with MUNO, ^c^*P*<0.05

**Fig. 2:**
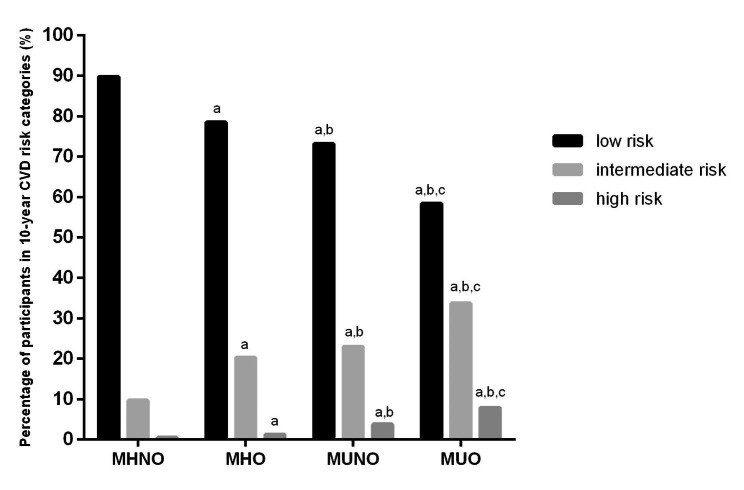
Percentages of 10-CVD risk categorise compared among different phenotypes. Compared with MHNO, ^a^*P*<0.05; Compared with MHO, ^b^*P*<0.05; Compared with MUNO, ^c^*P*<0.05

Figure 1 indicated that the age-adjusted mean baPWV values significantly increased in the MHNO phenotype compared to the MUO phenotype in the overall population (1234.08 ± 215.99 vs. 1283.57 ± 213.77 vs. 1423.29 ± 240.72 vs. 1446.19 ± 233.65 cm/s, *P* < 0.001). This phenomenon was also observed in men (1300.62 ± 202.51 vs. 1303.16 ± 201.40 vs. 1444.46 ± 231.24 vs. 1437.69 ± 225.67 cm/s, *P* < 0.001) and women (1194.56 ± 214.07 vs. 1246.03 ± 231.19 vs.1402.35 ± 248.08 vs. 1470.67 ± 253.77 cm/s, *P* < 0.001). In addition, the percentages of individuals with intermediate and high CVD risk were significantly higher in the MUO and MUNO phenotypes than in the MHNO and MHO phenotypes (Fig. 2).

### Relationship between elevated baPWV and different phenotypes by multivariate logistic regression analysis

When MHNO was used as a reference, the MUNO (*OR* = 2.843, *95% CI* 2.458–3.287, *P* < 0.001) and MUO (*OR* = 2.757, *95% CI* 2.420–3.142, *P* < 0.001) phenotypes were independently associated with elevated baPWV after adjusting for multiple risk factors (Table 2). However, after adjusting for traditional risk factors, there was no relationship between MHO and the risk of elevated baPWV.

**Table 2:** Odds ratios (95% CI) of high baPWV across weight and metabolic status categories in all participants

Model	MHNO	MHO		MUNO		MUO	
		*OR* (95%*CI*)	*P*	*OR* (95%*CI*)	*P*	*OR* (95% *CI*)	*P*
1	1.00 (ref)	1.399(1.225–1.597)	< 0.001	4.143(3.662–4.687)	< 0.001	4.445(4.004–4.936)	< 0.001
2	1.00 (ref)	1.147(0.984–1.336)	0.079	2.843(2.458–3.287)	< 0.001	2.757(2.420–3.142)	< 0.001

Model1: unadjusted

Model2: adjustment for age, sex, smoking, WBCC, Neutrophil count, TC, LDL-C, HbA1c and uric acid

### Relationship between the 10-year CVD risk and different phenotypes by multivariate logistic regression analysis

Table 3 showed the ORs for intermediate or high CVD risk according to different phenotypes. The OR values were higher in other phenotypes when compared with the MHNO phenotype: 1.698 (95% CI 1.471–1.961, *P* < 0.001) for the MHO group, 2.198 (*95% CI* 1.893–2.553, *P* < 0.001) for the MUNO group, and 3.244 (*95% CI* 2.885–3.685, *P* < 0.001) for the MUO group, after adjusting for confounding variables.

**Table 3:** Odds ratios (95% CI) of 10-year CVD risk across weight and metabolic status categories in all participants

Model	MHNO	MHO		MUNO		MUO	
		*OR* (95%*CI*)	*P*	*OR* (95% *CI*)	*P*	*OR* (95% *CI*)	*P*
1	1.00 (ref)	2.417(2.118–2.759)	< 0.001	3.225(2.816–3.695)	< 0.001	6.292(5.627–7.035)	< 0.001
2	1.00 (ref)	1.698(1.471–1.961)	< 0.001	2.198(1.893–2.553)	< 0.001	3.244(2.885–3.685)	< 0.001

Model1: unadjusted

Model2: adjustment for WBCC, Neutrophil count, LDL-C, HbA1c and uric acid

### The association of elevated baPWV with BMI in metabolically healthy and metabolically unhealthy participants by binary logistic regression analysis

In metabolically healthy participants, BMI (OR = 1.067, *95% CI* 1.041–1.092, *P* < 0.001) was independently correlated with elevated baPWV after adjusting for MetS components, including SBP, TG, HDL-C and FPG. However, the association between BMI (OR = 1.009, *95% CI* 0.991–1.027, *P* = 0.313) and elevated baPWV was nonsignificant in metabolically unhealthy participants.

### The association of the 10-year CVD risk with BMI in metabolically healthy and metabolically unhealthy participants by binary logistic regression analysis

In both metabolically healthy and metabolically unhealthy participants, BMI was significantly correlated with intermediate or high CVD risk, even after adjustment for MetS components, including SBP, FPG, TG and HDL-C. The OR value was 1.049 (*95% CI* 1.022–1.077, *P* < 0.001) for the metabolically healthy participants and 1.047 *(95% CI* 1.028–1.067, *P* < 0.001) for the metabolically unhealthy participants.

### The interaction of metabolic health status in the relationship between BMI and elevated baPWV

**Fig. 3:**
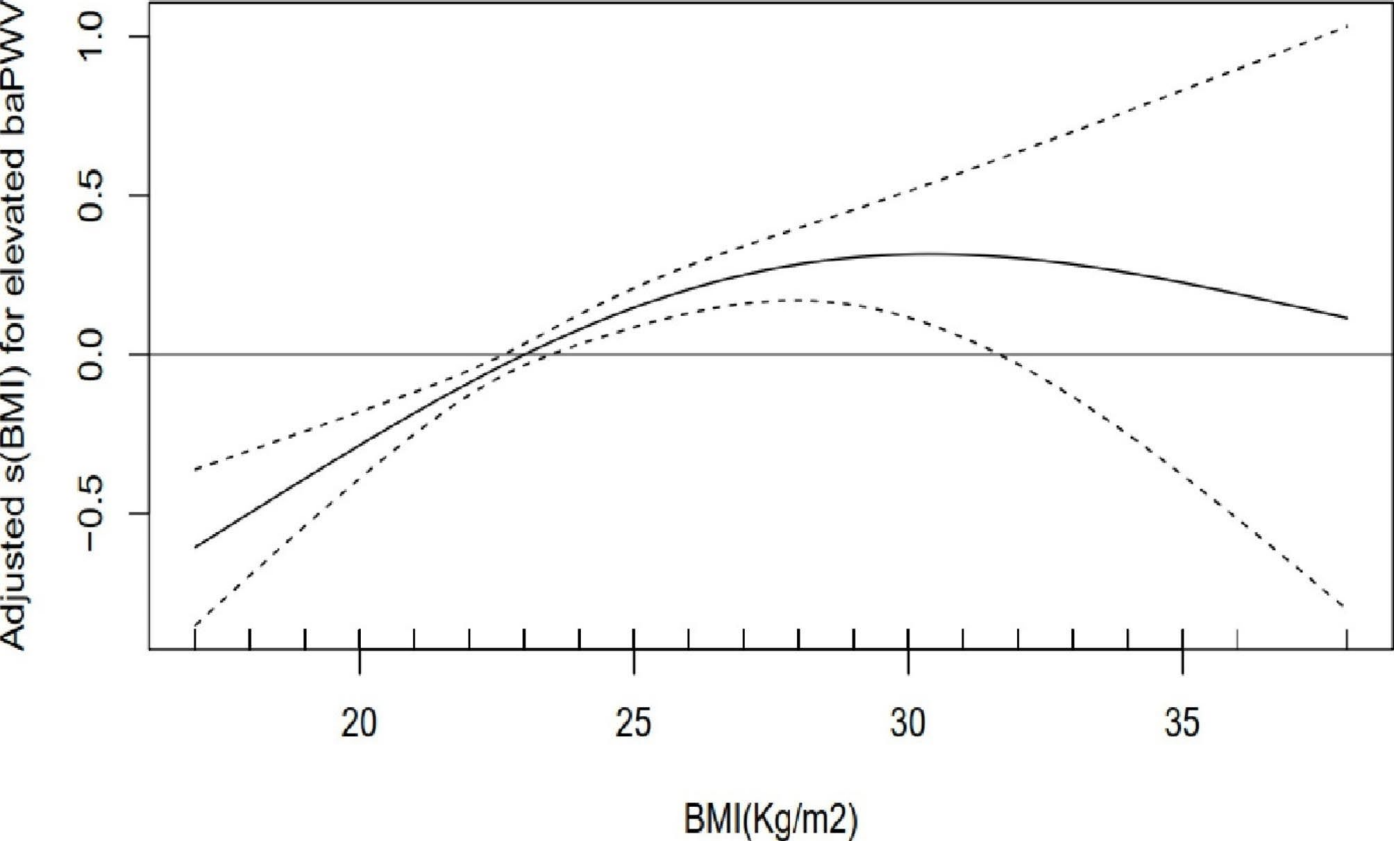
Dose–response relationship between BMI and elevated baPWV in metabolically healthy participants

**Fig. 4:**
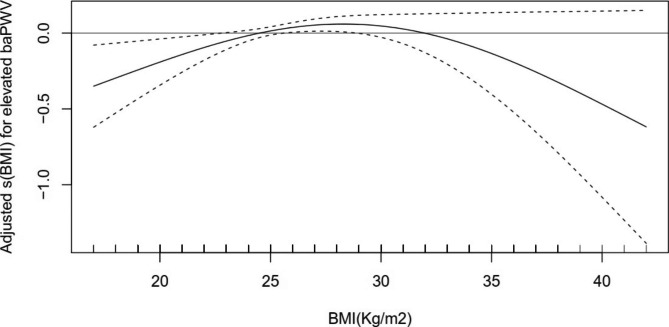
Dose–response relationship between BMI and elevated baPWV in metabolically unhealthy participants

Metabolic health status was an interaction factor between BMI and elevated baPWV in all study participants (*P* < 0.001). In metabolically healthy participants, BMI indicated a dose-dependent increase in the risk of elevated baPWV, with an adjusted OR of 1.007 (*95% CI* 1.004–1.010, *P* < 0.001) (Fig. 3). However, in metabolically unhealthy participants, the estimate for the relationship between elevated baPWV and BMI was nonsignificant (Fig. 4).

## Discussion

The present study showed that MUNO individuals exhibited significantly higher baPWV and 10-year CVD risk than MHO or MHNO individuals. Furthermore, the ORs for elevated baPWV and intermediate or high CVD risk in MUNO individuals were significantly higher than those in the MHO or MHNO groups, even after adjusting for confounding factors. However, there was no association between MHO and elevated baPWV after adjusting for classical CVD risk factors. Moreover, BMI was independently associated with elevated baPWV in metabolically healthy participants. The relationship was not present in metabolically unhealthy participants, for whom MetS itself was the dominant risk factor for arterial stiffness.

MetS is a worldwide epidemic that consists of abdominal obesity, elevated blood pressure, dyslipidemia and hyperglycemia. All components are correlated with an increased CVD risk. Interestingly, there is a unique condition of being a metabolically abnormal but not obese individual, referred to as MUNO. Compelling evidence has demonstrated that MUNO individuals have a high risk of CVD. The Nurses’ Health Study demonstrated that women with metabolically unhealthy normal weight and those who are overweight had a considerably higher CVD risk [5]. The Framingham Heart Study showed that MUNO participants had 1.95-fold and 1.92-fold higher odds of subclinical CVD and coronary artery calcification, respectively, than MHNO participants [16]. Stefan N et al. indicated that risks of all-cause mortality and/or cardiovascular events were 3-fold higher in subjects with MUNO than in MHNO individuals [17, 18]. BaPWV is a noninvasive method to evaluate arterial stiffness. Studies have indicated that baPWV is a simple and valuable predictor of cardiovascular mortality and CVD events [18, 19]. Although previous studies have demonstrated that MetS and its components are correlated with arterial stiffness [20, 21], there are limited data about the relationship between arterial stiffness and MUNO, especially in a large sample of Chinese adults. Because there is no definite cutoff value for elevated baPWV and the cutoff value is different between male and female populations, it is reasonable to define the sex-specific upper quartile of baPWV of the study population as the cutoff value of elevated baPWV. To the best of our knowledge, three studies investigated the relationship between MUNO and baPWV in a Chinese population, but the cutoff value of elevated baPWV was robust in two of the three studies [22, 23]. In addition, the study population was smaller than the present study [15]. Our study, which was conducted among a relatively high number of Chinese adults and adopted a reasonable cutoff value of elevated baPWV, showed that participants with MUNO had a significantly higher baPWV and odds ratio of elevated baPWV than participants with MHNO and MHO, consistent with previous studies [22, 23]. In addition, the Framingham risk score was used to assess the 10-year CVD risk in MUNO individuals. We also found that the percentages of individuals with intermediate and high CVD risk were significantly higher in the MUNO phenotype than in the MHNO and MHO phenotypes, consistent with the study of Adair KE et al. [24]. Moreover, the MUNO phenotype was significantly correlated with intermediate or high CVD risk after adjustment for confounding factors. These results implicate MUNO as an opportunity for the primary prevention of CVD. The mechanism by which MUNO is significantly related to high CVD risk is poorly understood. Convincing evidence suggests that visceral adipose tissue, which is a metabolically active and proinflammatory fat, might contribute to increased CVD risk [25]. BMI is commonly adopted as a useful indicator of general obesity, and it does not take into account body composition or the amount of body fat. Although MUNO participants may not meet the criteria for obesity as measured by BMI, they may carry more visceral fat and therefore be subject to a greater risk of CVD. On the other hand, individuals with MUNO had high oxidized LDL levels, unfavorable inflammation profiles and an increased risk of low muscle mass [26, 27], all of which were significantly correlated with CVD.

Recently, whether MHO increases the risk of CVD has been a debated topic in the field of medicine and public health. However, there is considerable controversy surrounding this issue. Evidence arising from longitudinal prospective studies has revealed that MHO is not a stable state and that some MHO individuals will transition to the metabolically unhealthy phenotype [10, 28]. A prospective study including 6220 Chinese adults indicated that there was no relationship between baseline MHO and the incidence of subclinical atherosclerosis. However, those who transitioned to MHO had an increased subclinical atherosclerosis risk [23]. A prospective cohort study in a general Chinese population showed that even maintaining the MHO phenotype over time was correlated with a higher CVD risk [29]. Cho YK et al. demonstrated that individuals in the baseline MHO group had a higher risk of CVD events than those in the MHNO group. Meanwhile, individuals who transitioned from MHO to MUO phenotypes had a higher risk of CVD events and all-cause mortality than stable MHO individuals [30]. Itoh H et al. found that MHO individuals had a higher prevalence of carotid plaques than non-obese individuals, indicating the potential significance of MHO in the development of subsequent CVD [31]. However, the Framingham Heart Study indicated that the MHO phenotype was not associated with prevalent subclinical CVD when compared with the MHNO phenotype [16]. Subsequently, the 2015–2016 National Health and Nutrition Examination Survey in the USA confirmed that there was no difference in CVD risk between the MHO phenotype and MHNO phenotype [24]. Similarly, there are limited data about the association of arterial stiffness with the MHO phenotype, especially in a large Chinese population. In this study, we demonstrated that baPWV values were significantly higher in participants with MHO than in participants with MHNO. However, there was no relationship between MHO individuals and the risk of elevated baPWV after adjustment for metabolic parameters, including SBP, lipid profile and blood glucose. We speculated that metabolic status may be a strong confounder in the obesity–elevated baPWV association. Hence, we further used generalized linear models to explore the metabolic status-interaction between BMI and elevated baPWV. Beyond that, we also found that BMI was significantly associated with elevated baPWV, even after adjusting for MetS components, in metabolically healthy participants. However, this association was not present in metabolically unhealthy participants. Consistent with our results, a growing body of evidence has demonstrated that obesity is not an independent risk factor for CVD and mortality when obesity and metabolic disorders are considered together [32]. Therefore, obesity is likely a major primary cause of increased CVD risk only in the metabolically healthy population.

Another major finding of the present study was that the percentages of individuals with intermediate and high CVD risk were significantly higher in the MHO phenotype than in the MHNO phenotype. We also found that the MHO phenotype was positively associated with increased intermediate or high CVD risk, even after adjustment for confounding factors. Moreover, this association existed in both metabolically healthy and metabolically unhealthy participants. The Framingham risk score was calculated based on sex, age, SBP, tobacco smoking, TC and HDL-C. Obesity was significantly correlated with most components of the Framingham risk score. However, our findings were not consistent with the study of Adair KE et al. [24]. The different definitions of metabolic health and obesity may explain the different results.

This study has several limitations. First, this study was a cross-sectional study to investigate the association of MUNO and MHO individuals with arterial stiffness and 10-year CVD risk; however, a prospective study is more convincing than a cross-sectional study. Second, we could not collect data that affected obesity and metabolic status, such as the nutritional diet and exercise habits of the participants. Third, measured variables such as BMI are also limited because they do not take into account true body composition.

In conclusion, the present study has shown an increased arterial stiffness and 10-year CVD risk in individuals with the MUNO phenotype. However, after adjusting for other CVD risk factors, MHO individuals only had an increased 10-year CVD risk. In particular, a mediating effect of metabolic profile has been detected between arterial stiffness and BMI. These findings provide new evidence that decisions on the initiation of lifestyle interventions should not be based solely on BMI; rather, metabolic status seems to be even more important.

## Data Availability

The datasets used and/or analyzed during the current study are available from the corresponding author on reasonable request.

## Abbreviations

BMI: Body mass index
SBP: Systolic blood pressure
DBP: Diastolic blood pressure
WBCC: White blood cell count
FPG: Fasting plasma glucose
HbA1c: Glycated hemoglobin A1c
TC: Total cholesterol
TG: Triacylglyceride
LDL-C: Low-density lipoprotein cholesterol
HDL-C: High density lipoprotein cholesterol
MUNO: Metabolically unhealthy non-obesity
MHNO: Non-obese subjects without metabolic syndrome
MHO: Metabolically healthy obesity
MUO: Metabolically unhealthy obesity
CVD: Cardiovascular disease
baPWV: Brachial ankle pulse wave velocity
FRS: Framingham risk score
